# Supplementary material for: Validation Study of Novel Point-of-Care Tests for Infliximab, Adalimumab and C-Reactive Protein in Capillary Blood and Calprotectin in Faeces in an Ambulatory Inflammatory Bowel Disease Care Setting
Source: Diagnostics (Basel). 2023 May 12;13(10):1712. doi: 10.3390/diagnostics13101712 (PMC10217227; doi:10.3390/diagnostics13101712)
Supplement: Supplementary file 1 [file diagnostics-13-01712-s001.zip › diagnostics-2261815-supplementary.pdf]

Supplementary Table S1. Infliximab **o**utliers >10 µg/mL difference between IFX ELISA and IFX CWB POCT with IFX serum retest

Patient	IFX ELISA	IFX CWB POCT	Δ (ELISA - CWB POCT)	IFX serum POCT	Δ (ELISA - serum POCT)	Excluded due to suspected timing error
1	46.00	32.8	13.2	39.8	6.2	No
2	6.7	18.5	-11.8	NA	NA	Yes
3	17.00	31.00	-14	18.7	-1.7	Yes
4	32.00	54.3	-22.3	NA	NA	Yes
5	3.2	34.2	-31.0	4.1	-0.9	Yes
6	7.0	>77.2	-70.2	9.2	-2.2	No (outside assay range)

All values in µg/mL.
